# Free Monoterpene Isomer Profiles of *Vitis Vinifera* L. cv. White Wines

**DOI:** 10.3390/foods7020027

**Published:** 2018-02-21

**Authors:** Mei Song, Claudio Fuentes, Athena Loos, Elizabeth Tomasino

**Affiliations:** 1 Department of Food Science & Technology, Oregon State University, Corvallis, OR 97331, USA; mei.song@oregonstate.edu (M.S.); anthena.loos@oregonstate.edu (A.L.); 2 Department of Statistics, Oregon State University, Corvallis, OR 97331, USA; fuentesc@science.oregonstate.edu

**Keywords:** chiral, enantiomer fraction, MDGC-MS, discriminant analysis, general linear model

## Abstract

Monoterpene compounds contribute floral and fruity characters to wine and are desired by grape growers and winemakers for many white wines. However, monoterpene isomers, especially monoterpene enantiomers, have been little explored. It is possible to identify and quantitate 17 monoterpene isomers in 148 varietal wines from eight grape varieties; Chardonnay, Gewürztraminer, Muscat, Pinot gris, Riesling, Sauvignon blanc, Torrontes, and Viognier in two vintages by Headspace solidphase microextraction multidimensional gas chromatography mass spectrometry (HS-SPME-MDGC-MS). Results obtained from general linear models and discriminant analysis showed significant differences for the isomer profiles and enantiomer fractions among the eight grape varieties and four wine styles. The high *R*^2^ values from the fitted line show low variation in enantiomeric differences based on variety. These results provide an overview of the monoterpene isomers of wide varietal wines, and support that isomer profiles and enantiomer fractions could differentiate our wines by varietal and wine style.

## 1. Introduction

White wines are frequently characterized by floral and fruity aromas, and these characteristics are major factors in determining the white wine character and quality. Monoterpenes have been widely investigated, as they are known to contribute to the floral and fruity aromas that are typical to aromatic white wines [1]. Monoterpenes are secondary metabolites that are primarily derived from grapes and affected by fermentation. Therefore, monoterpene profiles, including linalool oxide, linalool, hotrienol, nerol oxide, and citronellol have been used for varietal and place of origin classification [2,3,4,5]. For instance, linalool, nerol, geraniol, terpineol, linalool oxide, rose oxide, and nerol oxide have been used to differentiate Muscat wines from different regions [6,7,8,9]. Terpene profile patterns have been shown to differentiate Germany Muscat wines from neutral white wines (e.g., Sylvaner) as well [3,10]. However, there is limited information on enantiomeric composition of monoterpenes in white wines, and the differences in isomers have the potential to greatly influence the aroma of these wines. Despite their importance, monoterpenes are generally minor compounds with trace concentrations in grapes and wines and quantitation for these isomers are very limited to date.

There is an increasing interest in the determination of the enantiomeric ratios of chiral compounds in food flavors, fragrances, and additives [11,12]. Monoterpene enantiomers possess different sensory characteristics, such as *R*-(−)-linalool, with a described aroma of woody or lavender, while *S*-(+)-linalool possessing sweet, petitgrain-like aroma [13,14]. These different enantiomers also have very different perception thresholds, *R*-(−)-linalool (0.8 µg/L) has the odor perception 10 times lower than *S*-(+)-linalool (7.4 µg/L) in air [15]. Therefore, wines with slight variations in monoterpene compositions, especially enantiomeric ratios, may result in very different aroma perception. 

The present study was designed to investigate the monoterpene isomers in white wines that are produced from eight grape varieties collected from different regions and two vintages. The main objective was to determine the important monoterpenes isomers and enantiomer fractions in varietal wines, including any effect due to wine style. The different profiles of isomers may be important to the sensory differences in wines and can be used by the industry as markers of varietal quality.

## 2. Materials and Methods 

### 2.1. Chemicals

Chemical standards of *S*-(−)-limonene (≥99.0%), *R*-(+)-limonene (≥99.0%), (−)-rose oxide (≥99.0%), furanoid linalool oxide (≥97.0%), *R*-(−)-linalool (≥98.0%), were used to check the elution order of the isomer. Linalool (≥97.0%), *R*-(+)-α-terpineol (≥97.0%), and *R*-(+)-β-citronellol (98.0%) were purchased from Sigma Chemical Co. (St. Louis, MO, USA). *S*-(−)-α-Terpineol (96.0%) was purchased from BOC sciences (Ramsey Road, Shirley, NY, USA). Nerol oxide (99.0%) was bought from ALFA chemistry (Waverly Avenue, Holtsville, NY, USA). D3-(±)-α-terpineol and d3-(±)-linalool (≥99.4%) were obtained from CDN Isotopes (Pointe-Claire, QC, Canada). D3-(±)-limonene was synthesized in the lab via diels-alder and wittig reactions [16]. Milli-Q water was obtained from a Millipore Continental water system (EMD-Millipore, Billerica, MA, USA). HPLC (High Performance Liquid Chromatography) grade ethyl alcohol was obtained from Pharmco-AAPER (Vancouver, WA, USA). Residual sugar content was analyzed in duplicate according to the revised Rebelein method [17].

### 2.2. Samples 

One hundred and forty eight commercial white wines from eight grape varieties; Chardonnay, Gewürztraminer, Muscat, Pinot gris, Riesling, Sauvignon blanc, Torrontes, and Viognier, from two consecutive vintages, 2012 and 2013, were collected from regions known for those varieties [18]. All of the wines were purchased between February 2015 and March 2016. The wines were sampled into three 40 mL amber vials with PTFE/Silicone-lined caps (SUPELCO, Bellefonte, PA, USA) and three 50 mL centrifuge tubes (VWR International Corp., Visalia, CA, USA) right after purchased and stored at −20 °C until instrument and residual sugar analysis, which started from April 2016. 

### 2.3. Sample Preparation

Samples were prepared according to Song et al. [16] with some modifications described as follows. Each wine (2 mL) was diluted 4.5-fold with milli-Q water (6.85 mL) in 20 mL amber glass vials (Restek Corporation, Bellefonte, PA, USA). An isotopically-labelled internal standard solution (150 μL) containing d3-*S*-(−)-limonene, d3-*R*-(+)-limonene, d3-*R*-(−)-linalool, d3-S-(+)-linalool, d3-(−)-α-terpineol, and d3-(+)-α-terpineol was added, followed by 4.0 g of sodium chloride (VWR/JT Baker, Visali, CA, USA). The SPME (Solid Phase Micro-Extraction) vials were tightly capped with 18 mm PTFE-lined screw caps (Millipore Sigma, St. Louis, MO, USA).

The standard curves containing each isomer and the internal standard were established using de-aromatized dry Chardonnay wine following the stable isotope dilution analysis method [19]. A number of white wines were investigated, but Chardonnay was found to be the most appropriate across all of the wine styles (data not shown). Dry Chardonnay wine contained less aroma compounds and residual sugar contents, which minimized the matrix effects on the monoterpenes [16]. Standard curves were run twice for every 32 samples to minimize any fiber effects [16].

### 2.4. Head Space Solid Phase Micro-Extraction Coupled with Multidimensional Gas Chromatography Mass Spectrometery (HS-SPME-MDGC-MS)

The three-phase divinylbenzene/carboxen/polydimethylsiloxan (DVB/CAR/PDMS) Stableflex SPME fiber (50/30 µm thick, 2cm long, 24 Ga, SUPELCO, Bellefonte, PA, USA) with Shimadzu heart cut-MDGC-MS fitted with a Shimadzu AOC-5000 plus auto-sampler was used to perform sample analysis. Prior to SPME fiber extraction, the samples were equilibrated with agitation for 20 min (5 s on, 2 s off) at 60 °C. They were then extracted for 50 min with no further agitation. The fiber was injected into the MDGC-MS for 5 min at 250 °C, followed by further conditioning in an NDL heater for 3 min at 250 °C. 

An Rtx-wax column (Restek Corporation, PA, USA) was applied in the first GC (gas chromatography). An Rt^®^-βDEXsm connected using a zero dead volume internal union (Valco Instruments Co. Inc., Houston Texas, USA) to a Rt^®^-βDEXse column (Restek Corporation, Bellefonte, PA, USA) in series in the second GC. Method parameters were altered from Song et al. [16], due to the inclusion of nerol oxide isomers, as follows. Injector temperature was at 250 °C. The column oven for the first GC was held at 65 °C for 2 min, and then increased to 80 °C at 8 °C/min, following ramp to 125 °C at 2 °C/min for 20 min, then further increased to 230 °C at 8 °C/min and held at this temperature for 10 min. The column oven for the second GC was held at 40 °C for 2 min, then increased to 95 °C at 5.0 °C/min, held for 45 min, followed by an increase of 2.0 °C/min to 100 °C for 10 min, then further increased to 130 °C at 4.0 °C/min for 4 min, and finally increased to 220 °C at 20.0 °C/min and held at this temperature for 3 min. Quantifier, qualifier ions and heart-cut timings in the first GC selected for monoterpene isomers can be found in Table S1.

### 2.5. Statistical Analysis

Collected data was analyzed using general linear models (GLM) and discriminant analysis (DA) to study the relationship between the monoterpene isomer profiles, grape varieties, and wine style. One way ANOVA and Tukey’s honest significant difference (HSD) were used to compare mean differences among groups. All of the analyses were performed using IBM SPSS statistic 20 (SPSS Inc., Chicago, IL, USA). 

## 3. Results

### 3.1. Varietal Wines

Eight white grape varieties were selected and sampled from various regions (Table 1). Samples were balanced for all of the grape varietals over regions and vintages except for Torrontes wines. Argentina is the most well-known growing region for this grape variety and wines from this varietal could not be found from other regions.

Wine style was categorized according to European regulation [20]. Wine with residual sugar content less than 4 g/L can be considered as “dry”; between 4 g/L and 12 g/L as “medium dry”; between 12 g/L and 45 g/L as “medium sweet”; and, above 45 g/L as “sweet” wine. As can be seen in Table 1, most of the Chardonnay, Pinot gris, Riesling, Sauvignon blanc, Torrontes, and Viognier wines were dry and medium dry; Muscat wines were sweet; and, Gewürztraminer wines were abundant in medium dry and medium sweet styles.

### 3.2. Monoterpene Content in Varietal Wines

Seventeen monoterpene isomers, found in common across all of the wines, were well separated in single MDGC-MS run (Figure S1A,B). Standards for rose oxide, linalool oxide, and nerol oxide were isomer mixtures due to a lack of commercial resources for the singe isomers. The enantiomeric ratio was determined by the peak area ratio from three MDGC-MS injections of each isomer standard. The elution order for these isomers was determined from previous research using the same column [21]. Of the monoterpenes quantitated, two were monoterpene hydrocarbons, *S*-(−)-limonene, and *R*-(+)-limonene; ten were monoterpene oxides, (2*R*,4*S*)-(+)-*cis*-rose oxide, (2*S*,4*R*)-(−)-*cis*-rose oxide, (2*R*,4*R*)-(−)-*trans*-rose oxide, (2*S*,4*S*)-(+)-*trans*-rose oxide, (2*R*,5*R*)-(+)-*trans*-linalool oxide, (2*R*,5*S*)-(−)-*cis*-linalool oxide, (2*S*,5*S*)-(−)-*trans*-linalool oxide, (2*S*,5*R*)-(+)-*cis*-linalool oxide, *S*-(−)-nerol oxide, and *R*-(+)-nerol oxide; five were monoterpene alcohols, *R*-(−)-linalool, *S*-(+)-linalool, *S*-(−)-α-terpineol, *R*-(+)-α-terpineol, and *R*-(+)-β-citronellol (Table 2).

Significant differences (*p* < 0.05) for all of the monoterpene isomer concentrations were found among the eight varieties (Table 2). Higher concentrations of total isomers were found in Muscat and Torrontes wines (>1.2 mg/L) compared to other varieties, and the monoterpene constitutes of these two varieties were very similar. The lowest concentrations of total isomers (<40 µg/L) were found in Chardonnay and Pinot gris wines, which also had similar monoterpene profiles.

Total monoterpene hydrocarbons, which contribute citrus-like aromas [22], were abundant in Muscat (18.7 µg/L) and Torrontes (15.1 µg/L) wines. Rose oxides, described as rose-like aroma [14], had greater concentrations in Torrontes (4.0 µg/L), Gewürztraminer (1.2 µg/L), and Muscat wines (1.1 µg/L). Linalool oxide and nerol oxide isomers with green, leafy, spicy, floral characters [23,24], had greater quantities in wines from Torrontes (604.9 µg/L) and Muscat (405.2 µg/L), followed by Riesling wines (103.6 µg/L). Monoterpene alcohols with floral and fruity aromas [14,25,26] showed highest concentrations in Muscat (779.9 µg/L) and Torrontes (636.6 µg/L) wines. 

**Figure 1:**
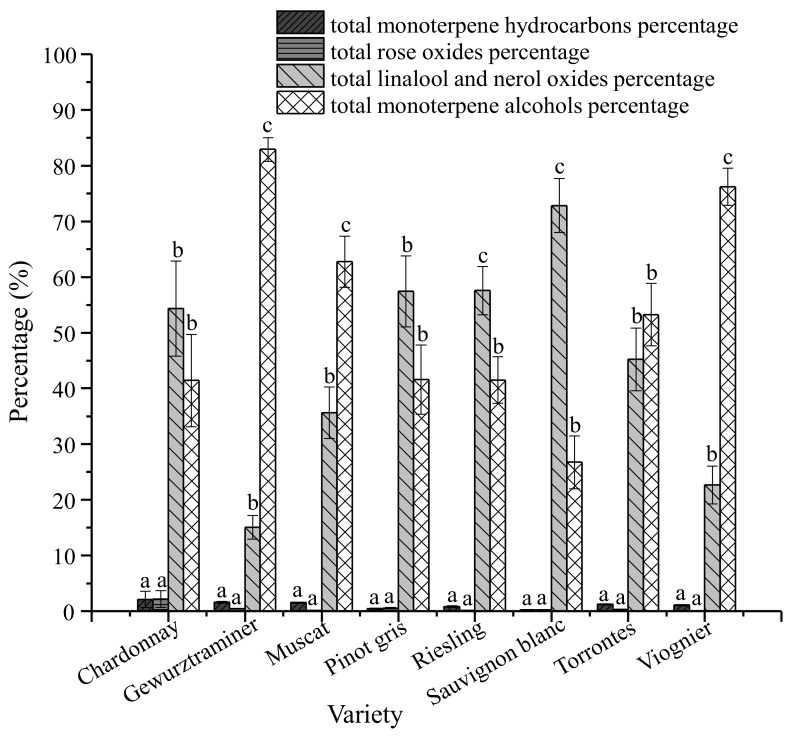
Clustered bar for isomers percentages in each varietal wine.

Not only did the isomer concentrations differ for all eight varieties, the monoterpene isomers with the greatest concentration in each varietal wine also differed (Figure 1, *p* < 0.05). Total monoterpene alcohols were dominant in Gewürztraminer, Viognier, Muscat, and Torrontes wines. Total linalool oxides and nerol oxide isomers had higher percentage in Sauvignon blanc, Pinot gris, Riesling and Chardonnay wines.

Means with the same letter are not significantly different from each other in each varietal wine (Tukey’s HSD, α = 0.05).

All of the wines were selected from different wineries, grape varieties, and regions, and were made in different ‘styles’ with varied amount of residual sugar. In order to determine any varietal and style effects that may influence monoterpene content, we considered a GLM procedure to model the monoterpene isomer profiles in wines in terms of two factors (no vintage effect was found, data not shown): grape variety and wine style. Due to restrictions with the degrees of freedom, additive models were used for most of the isomers, with the exception of the (2*R*,4*S*)-(+)-*cis*-rose oxide, (2*S*,5*R*)-(+)-*cis*-linalool oxide, and *S*-(−)-nerol oxide isomers, for which we explicitly considered the variety-style interaction term. The results showed that the mean concentration of each isomer had significant differences among the eight grape varieties and four wine styles (Table S3).

### 3.3. Classification of the Grape Variety and Wine Style Based on Monoterpene Isomer Concentrations

According to GLM results, grape variety, and style were significant factors for the model. For the discriminant analysis, the concentrations of 17 isomers were regarded as the independent variables, while the classes of grape varieties and styles were treated as categorical variables. The functions summarized in DA used a step-wise selection procedure based on the Wilks’ Lambda method.

**Figure 2:**
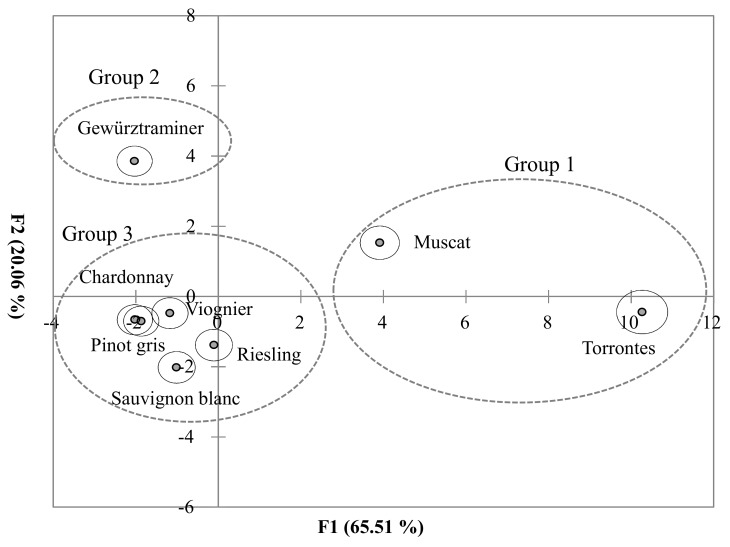
Discriminant plot of varietal wines on concentration of monoterpene isomers.

Two statistically significant discriminant functions were found, explaining 86% of the variability, with F1 and F2, contributing 66% and 20%, respectively (Figure 2). A separation between varietal wines was achieved in spite of the high variability coming from styles, resulting in three groupings. Muscat and Torrontes wines, grouped on the positive F1 direction, were clearly separated from the other wines (group 1). All of the compound vectors were in the positive F1 and F2 relating to the wines with the highest monoterpene isomer concentrations, Muscat and Torrontes (Figure S2). Linalool isomers were located in the same quadrant with Muscat wines, while rose oxide and nerol oxide isomers were located with Torrontes wines, so they could be the most important variables for the differentiation of these wines. All other wines were separated based on the F2 axis. Gewürztraminer had high positive scores on the F2 axis (group 2). Wines from Chardonnay, Pinot gris, Viognier, Riesling, and Sauvignon blanc were classified in one group that had negative scores along F2 (group 3).

Varietal wine scores were represented by centroids surrounded by 95% confident regions (the solid circle). Three groups were separated by the dashed circle.

**Figure 3:**
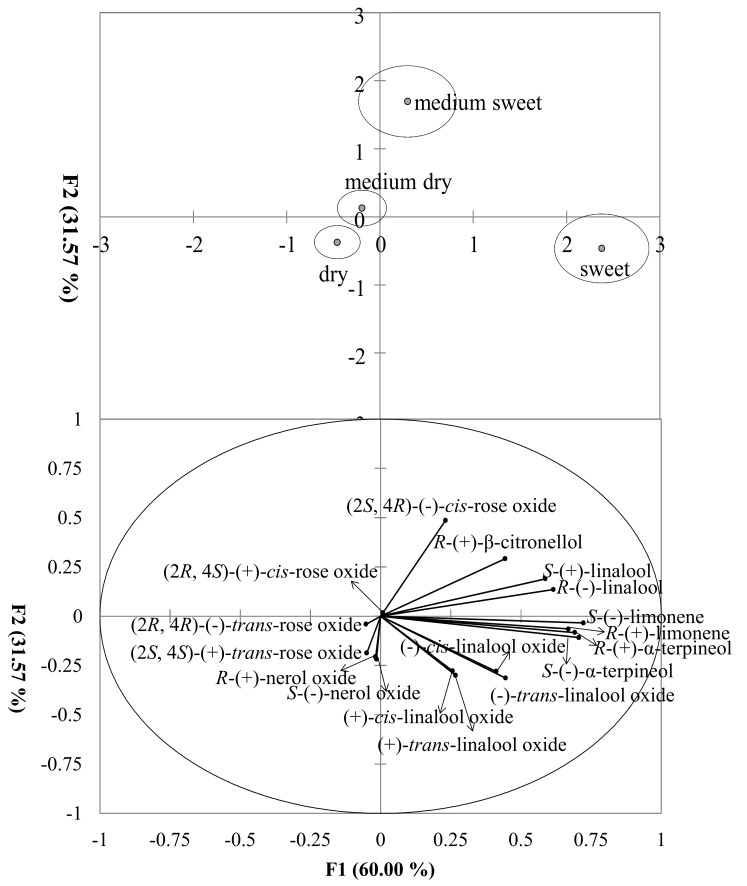
Discriminant plot of wine style on concentration of monoterpene isomers.

DA was also used to determine wine style based on monoterpene content. Three separated groups can be easily identified (Figure 3). Sweet wines were rich in monoterpene hydrocarbons, α-terpineol, and linalool oxide isomers and had the highest positive score in the F1 direction. Medium sweet wines contained greater concentrations of (−)-*cis*-rose oxide, *R*-(+)-β-citronellol, and linalool isomers with lower positive score along F1 axis. Medium sweet wines were separated from sweet wine by the F2 axis. Dry and medium dry wines had similar profiles with relative high concentrations of rose oxide and nerol oxide isomers. This was shown in the structure matrix (Table S4) as well.

Upper panel, Wines styles scores were represented by centroids surrounded by 95% confident regions. Bottom panel, all compound vectors were shown.

### 3.4. Enantiomer Fraction (EF) in Varietal Wines

Enantiomeric ratios have been investigated by other researchers [30,31]. However, when the concentration of the enantiomer in the ratio denominator was zero or non-detectable, the results were difficult to interpret. Our chosen method of investigating enantiomer differences, using enantiomer fraction (EF) [32], avoids this issue. EFs were calculated by dividing the earlier eluted enantiomer by the total enantiomers of each compound (enantiomer pairs) (Table 3, the elution order can be found in Figure S1), for example, *S*-(−)-limonene enantiomer fraction was calculated by the following Equation (1):

(1) $$S-(-)-\mathrm{limonene}\;(EF)=\frac{S-(-)-\mathrm{limonene}}{S-(-)-\mathrm{limonene}\;+\;R-(+)-\mathrm{limonene}}$$

As can be seen in Table 3, *S*-(−)-limonene EF was 0.39 in Chardonnay and Pinot gris, and 0.50 in Sauvignon blanc wines, which were significantly different from other varietal wines (*p* < 0.05). (2*R*,4*S*)-(+)-*cis*-Rose oxide EF was lower than 0.50 for all of the wines, which indicates that (2*S*,4*R*)-(−)-*cis*-rose oxide was the main *cis*-rose oxide enantiomer in all varietal wines. (2*R*,4*S*)-(−)-*trans*-Rose oxide EF was greater than 0.50 in all varietal wines and implies that (2*R*,4*R*)-(−)-*trans*-Rose oxide was the main *trans*-rose oxide enantiomer. (2*R*,5*R*)-(+)-*trans*-Linalool oxide and (2*R*,5*S*)-(−)-*cis*-linalool oxide had EFs greater than 0.50 in all varietal wines, except Viognier wines (0.37). *S*-(−)-nerol oxide and *R*-(+)-nerol oxide had very similar EF in all of the varietal wines. *R*-(−)-linalool and *S*-(−)-α-terpineol were both more than or equal to the corresponding enantiomer pair in all of the wines.

**Figure 4:**
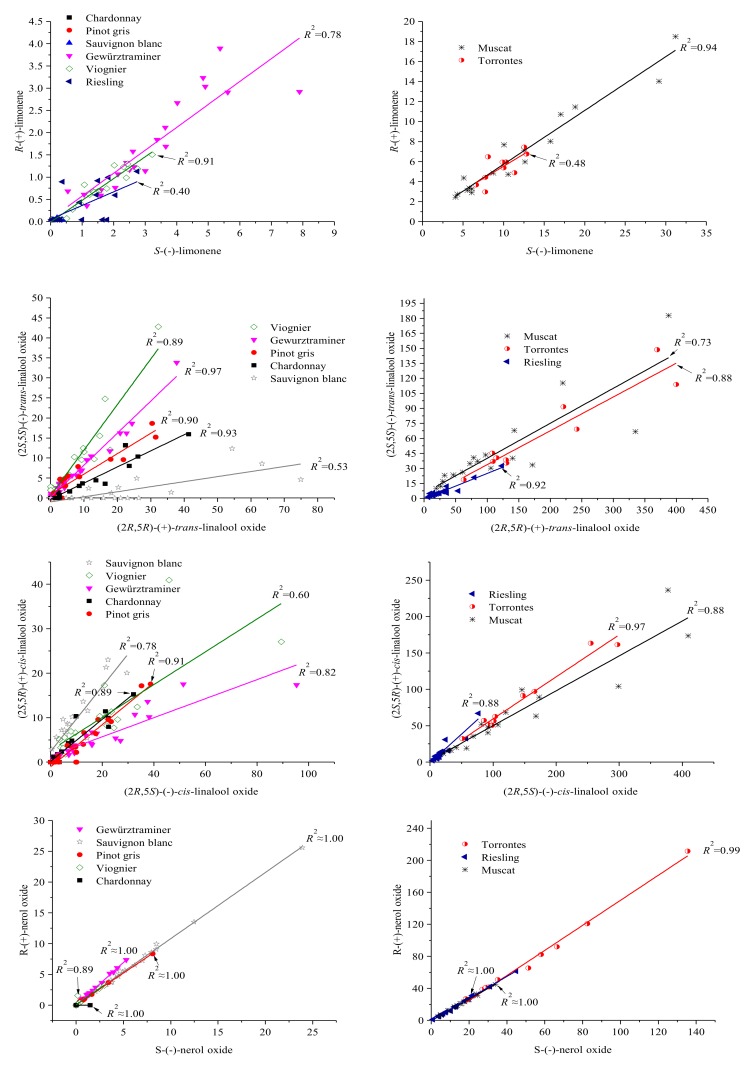
X-Y scatterplots of enantiomer pair concentrations (µg/L) in all varietal wines with fitted lines and adjusted *R*^2^.

The enantiomeric differences in the wines from different varieties can be seen vividly in Figure 4 and Figure S3. Varieties were separated into two scatterplots (Figure 4 and Figure S6) for the same enantiomer pair. The fitted lines were plotted for all wines from a single variety with some exceptions (Table 4), for example, Chardonnay, Pinot gris, and Sauvignon blanc wines in *S*-(−)-limonene vs. *R*-(+)-limonene scatterplot did not have fitted lines since there were only one or two data points in the scatterplot for these varieties.

The coefficient of determination *R*^2^ is the square of correlation between the actual value and the predicted value in a linear model, and can be interpreted as a measure of the variability in the response variable of enantiomeric ratios in each variety (higher *R*^2^ values indicating less variability of the response with respect to the fitted line). Most of the varieties showed high *R*^2^ values in enantiomer pairs, with *R*^2^ values greater than 0.8 (Table 4). Only one enantiomer pair had low *R*^2^ values (lower than 0.8) in Chardonnay, Pinot gris, and Viognier wines. The low *R*^2^ values in *S*-(−)-limonene pair of Riesling and Torrontes wines were all from medium sweet and sweet styles. The low *R*^2^ values in (2*R*,4*S*)-(+)-*cis*-rose oxide pair of Gewürztraminer and Riesling wines were mainly from the variations caused in the wines with higher monoterpene concentrations, for example, the large variation in Gewürztraminer was from concentrations of (2*R*,4*S*)-(+)-*cis*-rose oxide or (2*S*,4*R*)-(−)-*cis*-rose oxide higher than 0.8 µg/L. Most of *trans*-rose oxide enantiomers in Gewürztraminer wines, *trans*-linalool oxide enantiomers in Sauvignon blanc wines, α-terpineol enantiomers in Chardonnay, Pinot gris, and Sauvignon blanc wines were undetectable, therefore the variations (low *R*^2^ values) were caused by the concentrations from two groups in single variety. One group was the non-detectable enantiomers with concentrations assigned to LOD(limit of detection)/2; the other group was detectable isomers with higher concentrations.

The difference between enantiomer fractions among wines from different varieties can be seen from the slope of the fitted line in the X-Y scatterplots (Table 4). The slopes were very close for all or most of varietal wines in *S*-(−)-limonene, *S*-(−)-nerol oxide, *R*-(−)-linalool, or in *S*-(−)-α-terpineol scatterplots, which implied that the EF was similar in these enantiomer pairs for all varietal wines. However, the EF was quite different in rose oxide and linalool oxide enantiomer pairs, e.g., (2*R*,5*R*)-(+)-*trans*-linalool oxide.

In the same enantiomer pair, such as *S*-(−)-limonene vs. *R*-(+)-limonene, varietal wines were separated into two scatterplots based on the concentration range. The other four enantiomer pairs were shown in Figure S3.

### 3.5. Classification of the Grape Variety and Wine Style Based on Enantiomer Fractions

**Figure 5:**
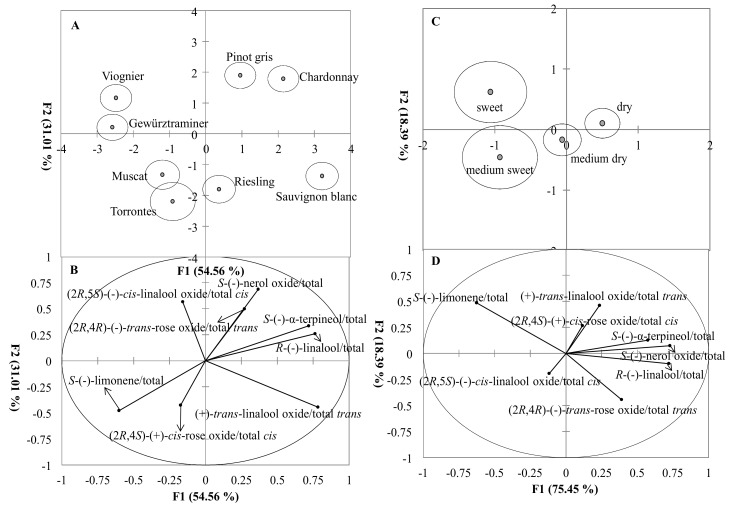
**Discriminant plot of grape variety and wine style on monoterpene enantiomer fractions**. Panel **A**, varietal wine scores were represented by centroids surrounded by 95% confident regions (the solid circle). Panel **B**, all enantiomer fractions vectors were shown based on varietal wines. Panel **C**, wine styles scores were represented by centroids surrounded by 95% confident regions. Panel **D**, all enantiomer fractions vectors were shown based on wine style.

The classification of varietal wines using EF calculation was different than by isomer concentrations (Figure 5A,B). The 86% of variability was explained by two statistically significant discriminant functions, with F1 and F2 contributing 55% and 31%, respectively. Muscat, Torrontes, and Riesling wines had similar EF profiles that are found in the lower positive or negative F1 direction with *S*-(−)-limonene and (2*R*,4*S*)-(+)-*cis*-rose oxide EFs as the important variables. Gewürztraminer and Viognier wines were separated from Muscat and Torrontes wines along the F2 axis, with profiles that were slightly different and characterized by (2*R*,5*S*)-(−)-*cis*-linalool oxide EF. Chardonnay and Pinot gris wines were clustered in the positive F1 and F2 direction with (2*R*, 4*R*)-(−)-*trans*-rose oxide, *S*-(−)-nerol oxide, *S*-(−)-α-terpineol, and *R*-(−)-linalool enantiomer fraction as important variables (Figure 5A,B). Sauvignon blanc wines located in the positive F1 direction with high positive scores, therefore (2*R*,5*R*)-(+)-*trans*-linalool oxide EF could be important variable to separate this varietal wine from others (Figure 5 B).

Style classification based on EFs for sweet and medium sweet wines were mainly distributed in the F1 function (75% of the variability) with high negative scores (Figure 5C). *S*-(−)-limonene EF were the separating variables (Figure 5D). Dry wines were located in the opposite direction along the positive F1 axis, with most of the EFs as the important variables. Medium dry wines were separated in the middle of F1 axis from other styles by (2*R*,5*S*)-(−)-*cis*-linalool oxide EF (Figure 5D).

## 4. Discussion

Monoterpene concentrations varied substantially between varietal wines. The total concentration of monoterpenes in Muscat and Torrontes wines were about four times greater than in Gewürztraminer wines, six times greater than in Riesling and Viognier wines, and over 30 times greater than in Chardonnay and Pinot gris wines (Table 2). In addition, limonene and nerol oxide were newly reported in these varietal wines. Similar results have been found when investigating free terpene concentrations of linalool, α-terpineol, citronellol, and linalool oxides [33,34], Muscat monoterpene content was 10 times higher than in other varieties, including Chardonnay, Gewürztraminer, and Riesling [34]. Recent studies found that rose oxide was highly correlated with Muscat and Gewürztraminer aroma [35,36]. Ong et al. reported higher *cis*-rose oxide contents in Gewürztraminer wines as compared to our study in older vintages, 1994, 1995, and 1997 [37]. Moreover, (2*S*,4*R*)-(−)-*cis*-rose oxide was found as the main stereoisomer with high enantiomeric ratios (higher than 90%) in all *V. vinifera* L. cv. Morio-Muskat must [38]. In our study, the largest concentrations of rose oxide were found in Torrontes wines, followed by Gewürztraminer and Muscat wines (Table 2). However, lower enantiomeric differences (EF: 0.23–0.49) of (2*R*,4*S*)-(+)-*cis*-rose oxide vs. (2*S*,4*R*)-(−)-*cis*-rose oxide was detected in our study as compared to Luan et al. 2006 (Table 3). This may be due to fermentation differences. (2*S*,4*R*)-(−)-*cis*-Rose oxide is formed through a reduction pathway during yeast fermentation and which may alter the enantiomeric ratio, thus forming more (2*R*,4*S*)-(+)-*cis*-rose oxide [39].

Low monoterpene concentrations in Chardonnay wines found in this study are in agreement with other research [40,41,42]. Specifically, that monoterpene content of Chardonnay must and juice makes up only 5% of the free volatiles [40,41,42]. This was anticipated as Chardonnay is considered more a neutral white wine grape and winemakers use oak and other techniques to increase the wines complexity [43,44].

The dominant monoterpene compositions differed among varietal wines. Our research shows that total monoterpene alcohols were abundant in Gewürztraminer, Viognier, Muscat, and Torrontes wines (Figure 1). Monoterpene alcohols play an important role in the aroma of Muscat and other aromatic grape varieties, and are thought to contribute to the fruity and floral notes [36,45]. Other researchers found that linalool was abundant in Muscat grapes with highly distinctive fruit character [7,46,47,48]. Linalool and citronellol have often been considered as important contributors to the aroma of Gewürztraminer, known for its floral, spicy notes [49]. Linalool oxide and nerol oxide were dominant isomers in Sauvignon blanc, Pinot gris, Riesling, and Chardonnay wines (Figure 1). Other studies showed little to no terpene content in Chardonnay, Sauvignon blanc, and Pinot gris wines [8]. Our results, however, show that these wines are abundant in linalool oxide and nerol oxide isomers. These differences in monoterpene isomers may be due to different biosynthesis pathways in aromatic and less aromatic grapes. .

Monoterpene profiles have been used to characterize grape varieties and varietal wines in other research [3,9,50]. Based on our results, the wines can be grouped into three classes for the varietal wines (Figure 2), very similar to the classification determined by Rapp et al. [10]. The most aromatic varietal wines included Torrontes and Muscat (group 1), Gewürztraminer wines (group 2), and the less aromatic varietal wines, including Chardonnay, Pinot gris, Riesling, and so forth (group 3). Interestingly, Riesling was grouped into the less aromatic wines in our study although Riesling has been generally considered as an aromatic grape variety.

Many terpene volatiles are direct products of terpene synthases in grapes [51], therefore the groupings based on monoterpene isomers could be linked to grape variety genetic information. The eight grape varieties selected are some of the most cultivated varieties in the world [8,18]. To date, the parentage information and genetic associations among these grape varieties are very limited. Torrontes possessed a distinct Muscat flavor and is the second most widely planted white grape cultivar in Argentina. It is very possible that Torrontes was the progeny of a cross between Muscat of Alexandria and Mission [52,53]. Consequently, Torrontes could contain some similar genetic information with Muscat that is responsible for monoterpene biosynthesis. This would explain our results, with similar monoterpene isomers in Torrontes and Muscat wines. Research using microsatellite markers has shown that Sauvignon blanc is progeny from Cabernet sauvignon [54]. Gewürztraminer originates from Italy and is a mutation of Sauvignon blanc [18]. Riesling originates from Germany, descended from the mother plant Heunisch Weiss [18]. Chardonnay and Pinot gris originate from France and are both progeny of Pinot noir [55]. Restriction fragment length polymorphism analysis showed that Chardonnay is 80% similar with Riesling, 76% with Sauvignon blanc, and 77% with Viognier [56]. To some extent these parental information do explain the groupings that are based on monoterpene isomers in this study. However, further parentage information related to these varieties cannot be traced from available resources.

The differences found in monoterpene content that cannot be explained by possible grape genetics may be linked to winemaking practices and known chemical stability at wine conditions [57,58]. Monoterpene formation can occur through different pathways, including grape biosynthesis [59], biotransformation by bacteria or fungi [60,61], alcoholic fermentation [62,63,64], and aging [65]. The low monoterpene content of Chardonnay wines may be due to de novo production of monoterpenes by *Saccharomyces cerevisiae* [66]. Further research in to the source of monoterpenes in neutral wines is needed to determine if monoterpene content is due to grape variety or yeast. All other varieties in this study had one or more monoterpenes at concentrations above those found by Carrau et al. [66], supporting the fact that monoterpenes originate from grapes. Additionally, the profiles of monoterpenes are known to change due to chemical rearrangement [67]. Polyols such as 3,7-dimethylocta-l,5-diene-3,7-diol, 3,7-dimethylocta-l,7-diene-3,6-diol, 3,7-dimethyloct-l-ene-3,7-diol, and 3,7-dimethyloct-lene-3,6,7-triol found in grape varieties can be non-enzymatically rearranged under conditions at grape juice pH of 3.2 [68]. The rearrangement results in formation of other monoterpenes, such as nerol oxide and furanoid linalool oxide, which are highly reactive [6].

It is well known that certain pairs of enantiomers possess different sensory characteristics that could alter aroma perception in wines. However, there have not been any studies to date on the EFs of monoterpenes in wines. The fitted lines in the X-Y scatterplots of enantiomer pairs had high *R*^2^ values for most of varietal wines (Table 4). It implied that the enantiomer difference of each compound is similar in wines from the single variety although these wines were collected from different wineries, regions, vintages and storage conditions. Our work showed that the majority of *R*^2^ values were at 0.8 or higher, suggesting that low variation in enantiomeric differences based on variety. The few low *R*^2^ values in the varietal wines may be attributed to several factors, including a low number of data points (e.g., *S*-(−)-α-terpineol pair in Chardonnay, Pinot gris and Sauvignon blanc wines), equipment error, or some extent of racemization occurred with confounding factors, such as different yeast strains, winemaking procedures, wine pH, and storage time [58]. Given that the majority of the *R*^2^ values (43 out of 58) were above 0.8 with 41 above 0.9, it seems unlikely that racemization or other differences due to storage or pH would affect the EF. Additional work would be required to completely rule out these factors.

The classification by EFs on varieties was different than by isomer profiles (Figure 2, Figure 5). Interestingly, most aromatic wines, such as Muscat, Torrontes, Gewürztraminer, Riesling, and Viognier were in one group, while neutral varietal wines, Chardonnay, and Pinot gris were in another group. It was reported that monoterpene synthases were not completely stereospecific in some species, therefore the accumulation of varied ratios of enantiomers occurred from a single enzyme [69]. The different EFs between aromatic grapes and neutral grape varieties may be related to the degree of stereo-specificity of monoterpene synthases or the differences in Chardonnay and Pinot gris may be due to yeast rather than grape origins of monoterpenes [66].

Wine style was also found to impact the isomer profiles and EF of these varietal wines (Figure 3, Figure 5). Monoterpene isomers in sweeter wines contained greater concentrations of linalool oxides, monoterpene alcohols, and hydrocarbons. In addition, despite the differences in grape varieties, sweeter wines (medium sweet and sweet style) had similar EFs. California dessert wines contained the greatest amount of these isomers (data not shown). Many dessert wines are fermented from overripe, shriveled/dried grapes. This “drying” process could provide an easier transfer of monoterpenes from skins to must during winemaking since monoterpenes are mostly abundant in grape skin [4]. In addition, the increasing concentrations of total bound terpenes and most individual terpenes in wine have been associated with the increase in grape maturity [49]. Research conducted on carrots showed a positive correlation between monoterpenes and sugar content [70], suggesting that monoterpenes may derive from their glycosides by acid-catalyzed reactions during aging [71,72].

## 5. Conclusions

Despite heterogeneity in regions, styles, vintages, and even winemaking techniques, varietal distinctiveness was supported by all of the wines studied based on isomer profiles and EFs. The research demonstrates the importance of isomer profiles and EFs to varietal white wines. This information is important for grape growers and winemakers that are attempting to produce wines with floral and fruity notes, or in specific distinctive styles. Further work is needed to determine the sensory contribution of the various monoterpene isomer profiles and EFs, and the origins of monoterpenes in more neutral varieties.

## Figures and Tables

**Table 1:** Distribution of varietal wines across, region of origin, vintage and wine style.

Variety	Region ^a^	Vintage	Style of Wine (Bottles)			
			Dry	Medium Dry	Medium Sweet	Sweet
Chardonnay	AU	2012	3			
		2013	2	2		
	OR	2012	4			
		2013	1	2	1	
	CAL	2012	3			
		2013	2	1		
Gewürztraminer	OR	2012		1	1	1
		2013	1	2		
	FR	2012		2	1	
		2013		1	4	
	NY	2012		1	1	
		2013		2	1	
	CAL	2012		1		
		2013		1		
Muscat	FR	2012		2		2
		2013	2	1		
	CAL	2012				2
		2013			1	2
	IT	2012				2
		2013				3
Pinot gris	OR	2012		4		
		2013	3	1		
	FR	2012		3		
		2013		3		
	IT	2012	2	2		
		2013	2	1		
Riesling	OR	2012		3		
		2013	1			
	FR	2012		3		
		2013	1	3		
	GR	2012			1	2
		2013		1	1	1
	AU	2013	1		1	
Sauvignon blanc	NZ	2012	3	1		
		2013	1	3		
	SA	2012	2			
		2013	3			
	FR	2012	3			
		2013	2	1		
Torrontes	ARG	2012	4	1		
		2013	4		1	
Viognier	FR	2012	3	1		
		2013	3			
	OR	2012	1			
		2013	1	3		
	CAL	2012	3	1		
		2013	3	1		

^a^ ARG: Argentina; AU: Australia; CAL: California; FR: France; GR: Germany; IT: Italy; NY: New York; NZ: New Zealand; OR: Oregon; SA: South Africa.

**Table 2:** Mean concentrations of isomers ^*^ ± standard error from different varietal wines (µg/L) using ANOVA with Tukey’s HSD (Honest Significant Difference) (α= 0.05) ^**^.

	Chardonnay	Gewürztraminer	Muscat	Pinot Gris	Riesling	Sauvignon Blanc	Torrontes	Viognier
*S*-(−)-limonene	0.03 ± 0.00 ^a^	3.17 ± 0.39 ^b^	11.96 ± 2.00 ^c^	0.03 ± 0.00 ^a^	0.87 ± 0.20 ^ab^	0.08 ± 0.02 ^a^	9.70 ± 0.67 ^c^	1.53 ± 0.19 ^ab^
*R*-(+)-limonene	0.04 ± 0.00 ^a^	1.69 ± 0.23 ^a^	6.78 ± 1.10 ^b^	0.04 ± 0.00 ^a^	0.32 ± 0.09 ^a^	0.05 ± 0.00 ^a^	5.39 ± 0.45 ^b^	0.74 ± 0.10 ^a^
Total hydrocarbon	0.07 ± 0.00 ^a^	4.86 ± 0.60 ^b^	18.74 ± 3.08 ^c^	0.07 ± 0.00 ^a^	1.19 ± 0.27 ^ab^	0.13 ± 0.02 ^a^	15.08 ± 1.05 ^c^	2.27 ± 0.29 ^ab^
(2*R*, 4*S*)-(+)-*cis*-rose oxide	0.02 ± 0.00 ^a^	0.20 ± 0.04 ^bc^	0.34 ± 0.09 ^c^	0.04 ± 0.01 ^ab^	0.07 ± 0.02 ^ab^	0.04 ± 0.01 ^ab^	0.80 ± 0.11 ^d^	0.06 ± 0.02 ^ab^
(2*S*, 4*R*)-(−)-*cis*-rose oxide	0.06 ± 0.00 ^a^	0.70 ± 0.17 ^bc^	0.50 ± 0.08 ^b^	0.09 ± 0.02 ^a^	0.07 ± 0.01 ^a^	0.07 ± 0.01 ^a^	0.85 ± 0.13 ^c^	0.06 ± 0.00 ^a^
(2*R*, 4*R*)-(−)-*trans*-rose oxide	0.00 ± 0.00 ^a^	0.25 ± 0.05 ^a^	0.20 ± 0.07 ^a^	0.01 ± 0.00 ^a^	0.04 ± 0.02 ^a^	0.02 ± 0.02 ^a^	1.57 ± 0.67 ^b^	0.00 ± 0.00 ^a^
(2*S*, 4*S*)-(+)-*trans*-rose oxide	0.00 ± 0.00 ^a^	0.05 ± 0.01 ^a^	0.11 ± 0.03 ^a^	0.00 ± 0.00 ^a^	0.02 ± 0.01 ^a^	0.01 ± 0.01 ^a^	0.74 ± 0.23 ^b^	0.00 ± 0.00 ^a^
Total rose oxide	0.08 ± 0.00 ^a^	1.19 ± 0.22 ^c^	1.14 ± 0.17 ^bc^	0.14 ± 0.03 ^a^	0.19 ± 0.05 ^ab^	0.15 ± 0.05 ^a^	3.96 ± 1.01 ^d^	0.12 ± 0.02 ^a^
(2*R*,5*R*)-(+)-*trans*-linalool oxide	8.53 ± 2.49 ^a^	9.70 ± 2.15 ^a^	120.55 ± 25.80 ^b^	7.11 ± 2.13 ^a^	32.22 ± 6.47 ^a^	23.63 ± 4.66 ^a^	188.60 ± 36.82 ^c^	7.51 ± 1.84 ^a^
(2*R*,5*S*)-(−)-*cis*-linalool oxide	6.98 ± 2.05 ^a^	20.79 ± 4.78 ^a^	136.21 ± 28.73 ^b^	11.77 ± 2.38 ^a^	19.78 ± 4.12 ^a^	8.82 ± 1.88 ^a^	139.63 ± 25.03 ^b^	19.32 ± 4.50 ^a^
(2*S*,5*S*)-(−)-*trans*-linalool oxide	3.14 ± 1.04 ^a^	7.93 ± 1.74 ^a^	47.32 ± 10.46 ^b^	4.48 ± 1.14 ^a^	7.78 ± 1.70 ^a^	2.02 ± 0.79 ^a^	63.78 ± 13.16 ^b^	9.13 ± 2.21 ^a^
(2*S*,5*R*)-(+)-*cis*-linalool oxide	3.48 ± 1.03 ^a^	5.86 ± 1.13 ^a^	67.42 ± 14.63 ^b^	4.53 ± 1.18 ^a^	14.75 ± 3.43 ^a^	8.86 ± 1.54 ^a^	82.39 ± 14.63 ^b^	9.80 ± 2.11 ^a^
*S*-(−)-nerol oxide	0.07 ± 0.07 ^a^	1.83 ± 0.30 ^ab^	14.41 ± 1.99 ^c^	0.80 ± 0.40 ^a^	12.27 ± 2.51 ^bc^	6.33 ± 1.17 ^abc^	53.35 ± 11.14 ^d^	0.33 ± 0.18 ^a^
*R*-(+)-nerol oxide	0.00 ± 0.00 ^a^	2.53 ± 0.41 ^ab^	19.30 ± 2.64 ^c^	0.88 ± 0.42 ^ab^	16.76 ± 3.47 ^bc^	6.86 ± 1.26 ^abc^	77.12 ± 17.49 ^d^	0.43 ± 0.21 ^a^
Total linalool and nerol oxide	22.20 ± 6.55 ^a^	48.65 ± 10.34 ^a^	405.20 ± 80.48 ^b^	29.58 ± 7.40 ^a^	103.55 ± 21.32 ^a^	56.50 ± 10.48 ^a^	604.87 ± 114.29 ^c^	46.51 ± 10.34 ^a^
*R*-(−)-linalool	0.77 ± 0.34 ^a^	57.49 ± 4.81 ^bc^	127.63 ± 36.88 ^d^	0.14 ± 0.13 ^a^	2.96 ± 1.02 ^ab^	0.54 ± 0.30 ^a^	63.35 ± 10.94 ^c^	29.85 ± 5.19 ^abc^
*S*-(+)-linalool	0.68 ± 0.32 ^a^	55.27 ± 5.97 ^b^	108.24 ± 31.80 ^c^	0.08 ± 0.07 ^a^	2.90 ± 1.05 ^a^	0.49 ± 0.29 ^a^	48.26 ± 10.08 ^ab^	24.90 ± 4.87 ^ab^
*S*-(−)-α-terpineol	1.80 ± 0.22 ^a^	56.35 ± 7.06 ^a^	276.36 ± 39.95 ^b^	5.63 ± 0.62 ^a^	30.31 ± 3.33 ^a^	10.13 ± 1.40 ^a^	264.04 ± 15.79 ^b^	49.02 ± 4.75 ^a^
*R*-(+)-α-terpineol	0.45 ± 0.16 ^a^	58.89 ± 7.62 ^b^	252.72 ± 34.37 ^c^	2.87 ± 0.74 ^a^	26.19 ± 3.29 ^ab^	3.28 ± 0.92 ^a^	248.49 ± 14.22 ^c^	45.36 ± 4.43 ^ab^
*R*-(+)-β-citronellol	1.67 ± 0.27 ^a^	17.55 ± 2.69 ^c^	14.98 ± 5.78 ^c^	1.21 ± 0.29 ^a^	0.44 ± 0.20 ^a^	0.70 ± 0.23 ^a^	12.51 ± 1.82 ^bc^	2.70 ± 0.46 ^ab^
Total alcohols	5.36 ± 0.98 ^a^	245.56 ± 22.89 ^b^	779.94 ± 143.43 ^c^	9.92 ± 1.08 ^a^	62.80 ± 8.03 ^ab^	15.13 ± 2.41 ^a^	636.64 ± 48.91 ^c^	151.84 ± 16.01 ^ab^
Total isomers	27.71 ± 6.36 ^a^	300.26 ± 30.65 ^a^	1205.02 ± 174.02 ^b^	39.71 ± 7.71 ^a^	167.73 ± 22.69 ^a^	71.90 ± 11.19 ^a^	1260.55 ± 81.84 ^b^	200.74 ± 20.32 ^a^

^*^ Means in the same row with the same letter are not significantly different from each other; ^**^ The value of all non-detectable compounds were assigned to a value of LOD/2 where LOD (Limit of Detection) is calculated from 3.3 × (standard deviation of y-intercepts of regression line, SD)/(slope of the regression line, b) [27,28,29]. The detailed information is in Table S2.

**Table 3:** Enantiomer fractions (EFs) ^*^ ± standard error found in different varietal wines determined by ANOVA and Tukey’s HSD (α= 0.05) ^**^.

	*S*-(−)-limonene/total	(2*R*,4*S*)-(+)-*cis*-rose oxide/total cis	(2*R*,4*R*)-(−)-*trans*-rose oxide/total trans	(2*R*,5*R*)-(+)-*trans*-linalool oxide/ total trans	(2*R*, 5*S*)-(−)-*cis*-linalool oxide/ total cis	*S*-(−)-nerol oxide/total	*R*-(−)-linalool/total	*S*-(−)-α-terpineol/total
Chardonnay	0.39 ± 0.00 ^a^	0.23 ± 0.00 ^a^	1.00 ± 0.00 ^c^	0.78 ± 0.02 ^bc^	0.77 ± 0.04 ^d^	0.59 ± 0.02 ^d^	0.79 ± 0.03 ^bc^	0.86 ± 0.05 ^b^
Gewürztraminer	0.66 ± 0.02 ^b^	0.25 ± 0.02 ^ab^	0.86 ± 0.03 ^bc^	0.49 ± 0.03 ^a^	0.75 ± 0.01 ^cd^	0.42 ± 0.00 ^ab^	0.52 ± 0.01 ^a^	0.49 ± 0.00 ^a^
Muscat	0.63 ± 0.01 ^b^	0.41 ± 0.05 ^cd^	0.76 ± 0.05 ^b^	0.70 ± 0.02 ^bc^	0.67 ± 0.01 ^bcd^	0.43 ± 0.00 ^ab^	0.54 ± 0.01 ^a^	0.52 ± 0.00 ^a^
Pinot gris	0.39 ± 0.00 ^a^	0.26 ± 0.02 ^ab^	0.98 ± 0.02 ^c^	0.65 ± 0.04 ^b^	0.79 ± 0.03 ^d^	0.52 ± 0.01 ^c^	0.85 ± 0.01 ^c^	0.75 ± 0.06 ^b^
Riesling	0.68 ± 0.05 ^b^	0.39 ± 0.05 ^bcd^	0.86 ± 0.07 ^bc^	0.80 ± 0.01 ^c^	0.59 ± 0.02 ^ab^	0.42 ± 0.00 ^ab^	0.71 ± 0.04 ^b^	0.55 ± 0.01 ^a^
Sauvignon blanc	0.50 ± 0.04 ^a^	0.28 ± 0.03 ^abc^	0.98 ± 0.02 ^c^	0.94 ± 0.02 ^d^	0.50 ± 0.05 ^a^	0.48 ± 0.00 ^bc^	0.81 ± 0.03 ^bc^	0.82 ± 0.04 ^b^
Torrontes	0.64 ± 0.01 ^b^	0.49 ± 0.02 ^d^	0.58 ± 0.07 ^a^	0.75 ± 0.01 ^bc^	0.63 ± 0.01 ^abc^	0.41 ± 0.00 ^a^	0.59 ± 0.02 ^a^	0.51 ± 0.00 ^a^
Viognier	0.68 ± 0.02 ^b^	0.33 ± 0.05 ^abc^	0.99 ± 0.01 ^c^	0.37 ± 0.04 ^a^	0.64 ± 0.02 ^bc^	0.52 ± 0.03 ^c^	0.57 ± 0.02 ^a^	0.52 ± 0.00 ^a^

^*^ Means in the same column with the same letter are not significantly different from each other. ^**^ The value of all non-detectable compounds were assigned to a value of LOD/2 where LOD is calculated from 3.3 × (standard deviation of y-intercepts of regression line, SD)/(slope of the regression line, b) [27,28,29]. The detailed information is in Table S2.

**Table 4:** The *R*^2^ value and slope for the fitted line in the X-Y scatterplots of Figure 4.

	*S*-(−)-limonene pair		(2*R*,4*S*)-(+)-*cis*-rose oxide pair		(2*R*,4*R*)-(−)-*trans*-rose oxide pair		(2*R*,5*R*)-(+)-*trans*-linalool oxide pair		(2*R*,5*S*)-(−)-*cis*-linalool oxide pair		*S*-(−)-nerol oxide pair		*R*-(−)-linalool pair		*S*-(−)-α-terpineol pair	
	*R^2^*	Slope	*R^2^*	Slope	*R^2^*	Slope	*R^2^*	Slope	*R^2^*	Slope	*R^2^*	Slope	*R^2^*	Slope	*R^2^*	Slope
Chardonnay	-	-	-	-	-	-	0.93	0.40	0.89	0.47	1.00	−1.6 × 10^−34^	0.97	0.94	0.35	0.45
Gewürztraminer	0.78	0.51	0.27	2.66	0.02	0.06	0.97	0.80	0.82	0.21	1.00	1.39	0.92	1.19	1.00	1.08
Muscat	0.94	0.54	0.27	0.50	0.80	0.42	0.73	0.35	0.88	0.48	1.00	1.32	0.99	0.86	1.00	0.86
Pinot gris	-	-	0.89	1.91	1.00	0.69	0.90	0.51	0.91	0.47	1.00	1.03	1.00	0.58	0.02	0.32
Riesling	0.40	0.31	0.32	0.29	0.51	0.31	0.92	0.25	0.88	0.78	1.00	1.37	0.97	1.02	0.86	0.92
Sauvignon blanc	-	-	0.71	0.74	1.00	0.59	0.53	0.13	0.78	0.73	1.00	1.07	0.99	0.95	0.30	0.38
Torrontes	0.48	0.49	0.61	0.97	0.92	0.33	0.88	0.34	0.97	0.58	0.99	1.56	0.85	0.86	0.97	0.88
Viognier	0.91	0.49	1.00	1.5 × 10^−17^	-	-	0.89	1.14	0.60	0.37	0.89	1.07	0.86	0.87	0.96	0.91

## Supplementary Materials

The following are available online at http://www.mdpi.com/2304-8158/7/2/27/s1, Table S1: Quantifier and qualifier ions selected for monoterpene isomers , Table S2: Limit of detection (LOD)/2 for monoterpene isomers (µg/L) and the number of wines with non-detectable isomer, Table S3: GLM multivariate test investigating varietal and style effects, Table S4: Structure matrix of variable correlations for wine styles classification by discriminant, Figure S1: A-B, Chromatograph of the monoterpene isomers in one Torrontes varietal wine from MDGC-MS. A-30 min to 63 min (small spectrum with selected ions of *S*-(−)-limonene, (2*R*, 4*R*)-(−)-*trans*-rose oxide, and (2*R*, 5*S*)-(−)-*cis*-linalool oxide attached due to co-eluted with other compounds), B-63 min to 87 min, Figure S2: Monoterpene isomers vector loadings for varietal wines classification by discriminant analysis, Figure S3: X-Y scatterplots of enantiomer pair concentrations (µg/L) in all varietal wines with fitted lines and adjusted *R*^2^.
