# Supplementary material for: Free Monoterpene Isomer Profiles of Vitis Vinifera L. cv. White Wines
Source: Foods. 2018 Feb 21;7(2):27. doi: 10.3390/foods7020027 (PMC5848131; doi:10.3390/foods7020027)
Supplement: Supplementary file 1 [file foods-07-00027-s001.pdf]

Table S1. Quantifier and qualifier ions selected for monoterpene isomers

Compounds	Ions chosen[1] (m/z)	Heart-cut timings in the first GC(min)
S-(-)-limonene	**107,**121,136	16.00-19.75
R-(+)-limonene	**107,**121,136	
(2*R*, 4*S*)-(+)-*cis*-rose oxide	**139,**69,83	26.00-29.75
(2*S*, 4*R*)-(-)-*cis*-rose oxide	**139,**69,83	
(2*R*, 4*R*)-(-)-*trans*-rose oxide	**139,**69,83	
(2*S*, 4*S*)-(+)-*trans*-rose oxide	**139,**69,83	
(2*R*,5*R*)-(+)-*trans*-linalool oxide	**94,**93,111	32.00-38.00
(2*R*,5*S*)-(-)-*cis*-linalool oxide	**94,**93,111	
(2*S*,5*S*)-(-)-*trans*-linalool oxide	**94,**93,111	
(2*S*,5*R*)-(+)-*cis*-linalool oxide	**94,**93,111	
S-(-)-nerol oxide	**152,** 96,83	
R-(+)-nerol oxide	**152,** 96,83	
R-(-)-linalool	**121,**93,136	41.00-44.50
S-(+)-linalool	**121,**93,136	
S-(-)-α-terpineol	**59,**81,121	53.00-56.25
R-(+)-α-terpineol	**59,**81,121	
R-(+)-β-citronellol	**95,**109,123	57.00-58.60

[1] Numbers in bold are quantifier ions

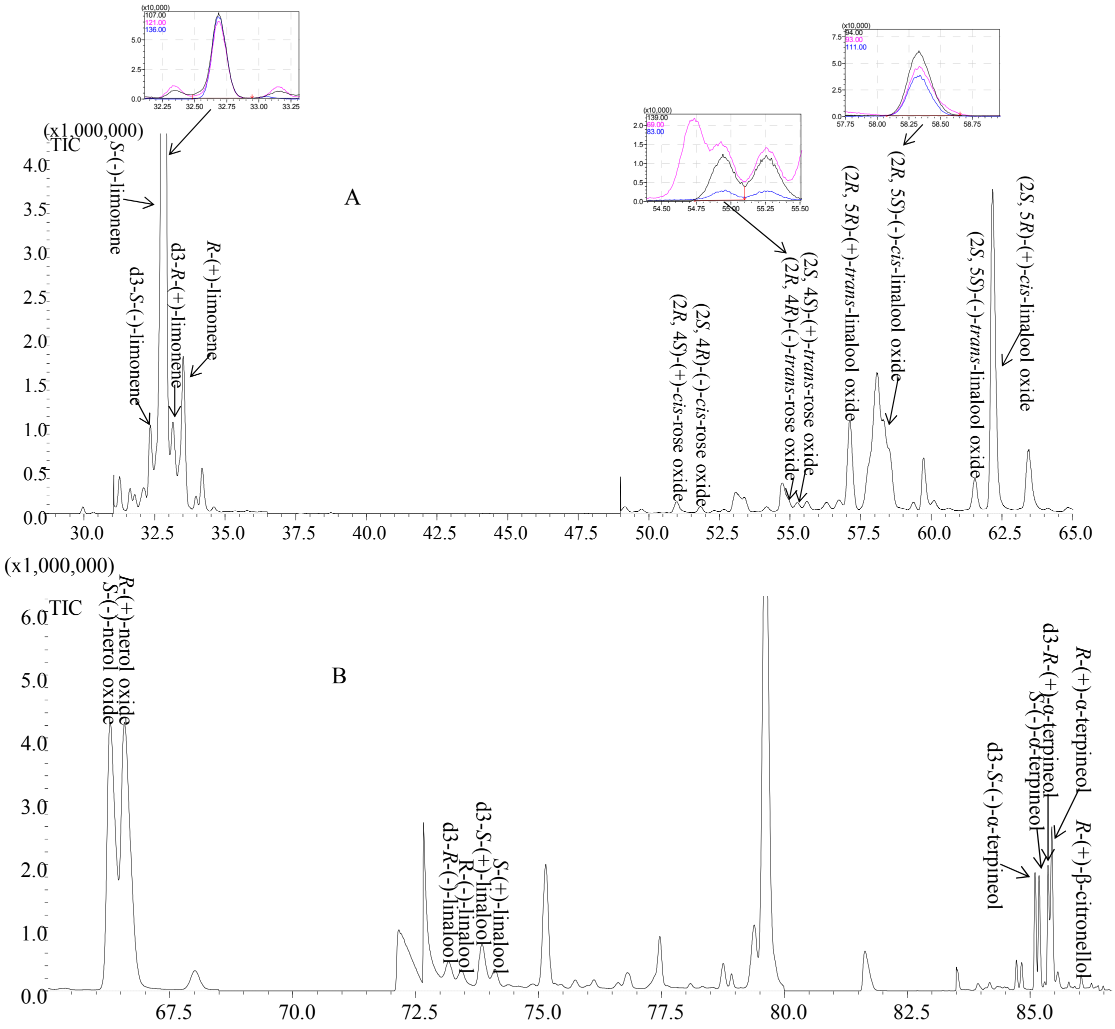

Figure S1. A-B, Chromatograph of the monoterpene isomers in one Torrontes varietal wine from MDGC-MS. A-30 min to 63 min (small spectrum with selected ions of *S*-(-)-limonene, (2*R*, 4*R*)-(-)-*trans*-rose oxide, and (2*R*, 5*S*)-(-)-*cis*-linalool oxide attached due to co-eluted with other compounds), B-63 min to 87 min.

Table S2. Limit of detection (LOD)/2 for monoterpene isomers (µg/L) and the number of wines with non-detectable isomer

	LOD/2	Chardonnay (21)*	Gewürztraminer (21)*	Muscat (17)*	Pinot gris (21)*	Riesling (19)*	Sauvignon blanc (19)*	Torrontes (10)*	Viognier (20)*
S-(-)-limonene	0.02808	21	0	0	21	4	13	0	1
R-(+)-limonene	0.04452	21	0	0	21	12	18	0	1
(2*R*,4*S*)-(+)-*cis*-rose oxide	0.01654	21	6	0	17	10	16	0	16
(2*S*,4*R*)-(-)-*cis*-rose oxide	0.05525	21	4	2	18	15	17	0	20
(2*R*,4*R*)-(-)-*trans*-rose oxide	0.00311	21	4	6	20	16	18	1	20
(2*S*,4*S*)-(+)-*trans*-rose oxide	0.00000	21	13	6	20	15	18	0	20
(2*R*,5*R*)-(+)-*trans*-linalool oxide	0.02211	7	1	0	4	0	1	0	18
(2*R*,5*S*)-(-)-*cis*-linalool oxide	0.01166	6	0	0	2	0	1	0	0
(2*S*,5*S*)-(-)-*trans*-linalool oxide	0.00649	10	0	0	7	0	11	0	0
(2*S*,5*R*)-(+)-*cis*-linalool oxide	0.00169	10	0	0	7	0	2	0	0
S-(-)-nerol oxide	0.00209	20	0	0	12	0	0	0	13
R-(+)-nerol oxide	0.00155	20	0	0	12	0	0	0	13
R-(-)-linalool	0.01450	16	0	0	20	11	16	0	1
S-(+)-linalool	0.00231	16	0	0	20	11	16	0	1

S-(-)-α-terpineol	0.00611	1	0	0	0	0	0	0	0
R-(+)-α-terpineol	0.01544	15	0	0	10	0	8	0	0
R-(+)-β-citronellol	0.00008	4	0	0	9	14	11	0	2

* The number in the bracket is the total number of wines for each variety.

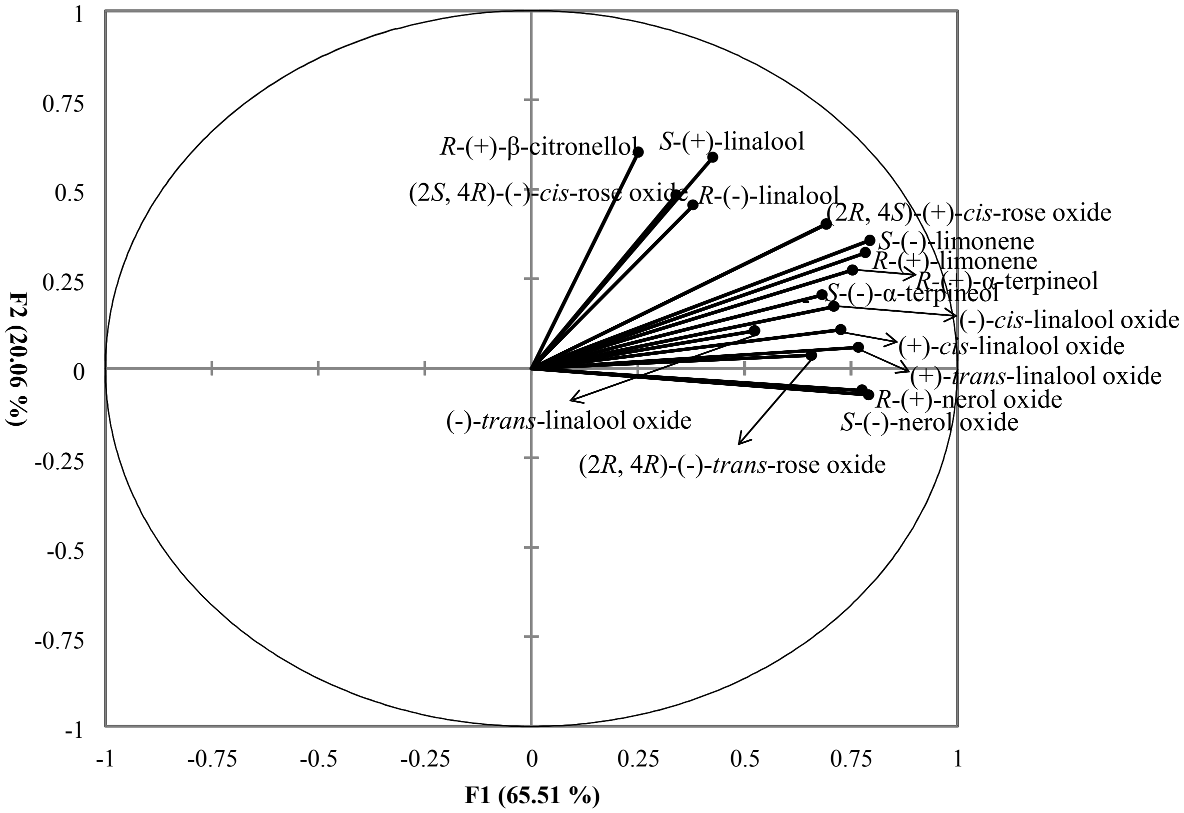

Figure S2. Monoterpene isomers vector loadings for varietal wines classification by discriminant analysis

Table S3. GLM multivariate test investigating varietal and style effects.

Effect	Test	F	Sig.
Intercept	Wilks' Lambda	30.802	.000
varietal	Wilks' Lambda	9.032	.000
style	Wilks' Lambda	1.852	.001

Table S4 Structure matrix of variable correlations for wine styles classification by discriminant analysis

	F1	F2	F3
S-(-)-limonene	0.723	-0.034	-0.158
R-(+)-limonene	0.670	-0.065	-0.091
(2*R*,4*S*)-(+)-*cis*-rose oxide	0.008	0.018	-0.242
(2*S*,4*R*)-(-)-*cis*-rose oxide	0.232	0.486	-0.317
(2*R*,4*R*)-(-)-*trans*-rose oxide	-0.052	-0.040	-0.384
(2*S*,4*S*)-(+)-*trans*-rose oxide	-0.049	-0.187	-0.354
(2*R*,5*R*)-(+)-*trans*-linalool oxide	0.267	-0.300	-0.200
(2*R*,5*S*)-(-)-*cis*-linalool oxide	0.412	-0.279	-0.092
(2*S*,5*S*)-(-)-*trans*-linalool oxide	0.445	-0.314	-0.266
(2*S*,5*R*)-(+)-*cis*-linalool oxide	0.256	-0.277	-0.114
S-(-)-nerol oxide	-0.014	-0.218	-0.283
R-(+)-nerol oxide	-0.018	-0.205	-0.298
R-(-)-linalool	0.615	0.135	-0.087
S-(+)-linalool	0.587	0.189	-0.041
S-(-)-α-terpineol	0.706	-0.108	-0.227
R-(+)-α-terpineol	0.692	-0.083	-0.235
R-(+)-β-citronellol	0.444	0.292	0.041

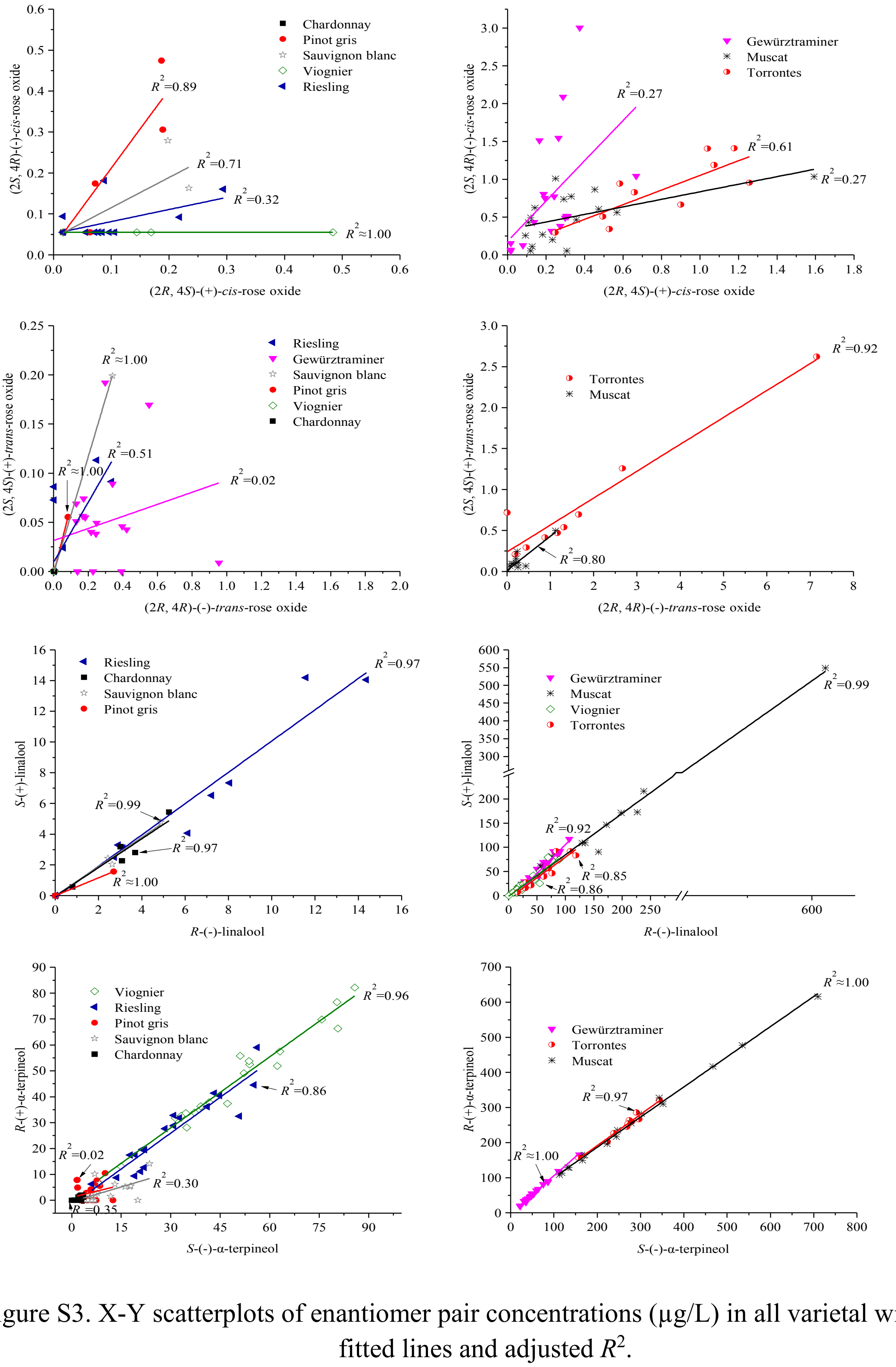

Figure S3. X-Y scatterplots of enantiomer pair concentrations (µg/L) in all varietal wines with fitted lines and adjusted R^2.
